# Colorectal Cancer and the Risk of Mortality Among Individuals with Suicidal Ideation

**DOI:** 10.3390/ijerph22060862

**Published:** 2025-05-30

**Authors:** Srikanta Banerjee, Jagdish Khubchandani, Stanley Nkemjika

**Affiliations:** 1 College of Health Sciences, Walden University, Minneapolis, MN 55401, USA; 2 College of Health, Education, and Social Transformation, New Mexico State University, Las Cruces, NM 88003, USA; jagdish@nmsu.edu; 3 Department of Psychiatry and Human Behavior, Thomas Jefferson University, Philadelphia, PA 19107, USA; stanley.nkemjika@waldenu.edu

**Keywords:** colorectal cancer, suicidal ideation, mortality risk

## Abstract

Suicide is a major public health problem that has grown at alarming rates in the last two decades. Colorectal cancer (CRC) is one of the most common causes of cancer deaths in the United States in both males and females. However, the influence of suicidal ideation (SI) on the association between CRC and risk of mortality has not been well examined. Methods: For this study, the 2005–2018 National Health and Nutrition Examination Survey (NHANES), a nationally representative survey of United States adults aged 20 years or older, was utilized. CRC survivorship was determined from self-reported data on CRC, and mortality was ascertained by linking the NHANES data with death files from the National Death Index up to December 2019. Suicidal ideation (SI) confirmation was based on a response to a survey question. Results: People with SI had a significantly higher rate (12.3% vs. 7.5%, *p* < 0.01) of mortality than those without SI. Also, individuals with CRC (2.4%) had a statistically significant higher rate of frequent SI (almost daily) than individuals without CRC (0.6%). Upon a mean follow-up of 7.5 years, more CRC survivors (34.6%) died than non-CRC survivors (7.6%). The adjusted HR was elevated HR = 5.4 among individuals who had CRC and SI but close to 1.0 among individuals who had a history of CRC alone after adjusting for demographic and health variables. Conclusions: In this first national study in the U.S., we found that SI and CRC combined had worse mortality outcomes than CRC alone. Additionally, people with CRC were more likely to experience frequent SI. Our findings underscore the importance of mental healthcare and psychological well-being promotion among individuals with chronic diseases and the high need for integrated care approaches that address both physical and mental health needs.

## 1. Introduction

Suicide rates have steadily increased in the U.S., with the year 2020 being the first time that suicide was among the top 10 causes of death in the nation. Between 2000 and 2020, the suicide rate increased from 10 per 100,000 to 14 per 100,000 [1,2,3]. The number of people who think about or attempt suicide in the U.S. is even higher, with more than 300 people experiencing suicidal ideation (SI) for every completed suicide [3,4]. In 2021, nonfatal self-harm and suicides were responsible for over USD 500 billion in costs from quality-of-life events, loss of worker productivity, and medical costs [5,6]. SI is associated with reduced functioning, lower quality of life, and adverse outcomes related to chronic diseases (e.g., cancer and cardiovascular diseases) [7,8,9]. The most common risk factors of suicide include mental illnesses, substance use disorders, chronic diseases, and life events, among others [8,9,10,11,12].

Among other causes of death in the U.S., cancer ranks in the top three. Recent statistics suggest that colorectal cancers (CRCs) are among the leading causes of cancer-related deaths and the most common types of cancers in adult Americans. Out of all anatomic sites, CRC is one of the most common cancers associated with suicide [11]. On average, the 5-year survival rate for these cancers is less than 70%, claiming more than 50,000 lives in the U.S. every year. CRC burden has been consistently increasing, and despite novel treatments and therapies, in the last decade, CRC rates have doubled among individuals younger than 55 years of age [13,14,15]. Cancer takes a huge toll on the biological, physiological, and psychological aspects of the overall health of survivors [16,17,18]. For example, several recent studies have suggested that individuals with CRC are more likely to develop mental illnesses, such as anxiety and depression. A recent meta-analysis indicated that individuals with CRC have a 51% increased risk of experiencing depression [16]. Furthermore, studies have also shown that colorectal patients with higher depressive and anxiety symptoms have greater pain, fatigue, inflammation, and potential for treatment failures [18,19]. Unfortunately, a significant proportion of adults in the U.S. with a chronic disease (e.g., CRC) do not receive timely, adequate, or quality mental healthcare. The burden of cancer, along with significant psychological distress, costs, and discomforts associated with treatments, becomes an ideal recipe for worsening mental health and suicidal ideation [18,19,20,21]. Few studies have examined the burden of SI among individuals with gastrointestinal cancers [20,21]. Furthermore, the impact of SI on the survival of individuals with CRC has not been explored. Therefore, the purpose of this national assessment was to assess the impact of SI on mortality risk among Americans with CRC.

## 2. Methods

### 2.1. Participants and Procedures

We used 7 waves (2005–2018, collected every year) of the National Health and Nutrition Examination Survey (NHANES), a program of the National Center for Health Statistics (NCHS), which was constructed to evaluate the health of adults in the United States using consolidated data from interviews and physical exams [22,23]. The analysis sample is representative of noninstitutionalized US adults ages 20 and older. The procedures and protocols for the NHANES were approved by the NCHS before data collection. The data are available for public use through the CDC website. We received additional ethical approval from the Walden University Institutional Review Board for data analysis.

### 2.2. Measures: Mortality

Vital status was determined using the Continuous (2005–2018) NHANES Public-Use Linked Mortality File, which provides vital status follow-up data on person from the date of their NHANES survey participation to their date of death or 31 December 2019, measured in months. Mortality was ascertained by the NCHS through probabilistic matching between NHANES participants and National Death Index (NDI) death certificate records [14,17,22,23]. Each possible NDI record match was assigned a probabilistic match score. The probabilistic match score was the sum of the weights assigned to each of the identifying data items used in the NDI record match, where the weights reflected the degree of agreement between the information on the submission record and the NDI death record. Only individuals with complete data to mortality or the end of the study period were included in this analysis, eliminating the need to censor data for the primary outcome of all-cause mortality.

### 2.3. Measures: Suicidal Ideation and CRC

Suicidal ideation among the respondents was assessed by one question in the NHANES data (i.e., “In the past two weeks, how often have you had thoughts of hurting yourself or believing that your life would be better off if you ended it?”, with the response options ranging from “Not at all”, to “several days”, “more than half the days”, and “almost every day”). For analytical purposes, all responses were categorized as either absent (no) or present at any frequency (yes) [20]. CRC status was determined using the medical conditions files from the NHANES surveys, where the participants were asked “Has a doctor or other health care professional ever told {you/SP} that {you/s/he} had cancer or a malignancy of any kind?” If yes, they were asked to specify the kind of cancer. The interviewers coded the cancer types reported, which were subsequently aggregated into categories. Colorectal cancer cases included the participants who reported being told by a doctor or healthcare professional that they had CRC. Sensitivity analyses were conducted for the CRC subgroups, which were coded analogously.

### 2.4. Measures: Comorbid Conditions

CVD was determined by self-reported diagnoses of coronary heart disease, angina, stroke, congestive heart disease, and heart attack. The respondents were also asked “{Have you/Has SP} ever been told by a doctor or other health professional that {you/s/he} had hypertension, also called high blood pressure?” Hypertension was defined as positive through self-reported data. Similarly, all respondents over the age of 20 were asked “Other than during pregnancy, have you ever been told by a doctor or other health professional that you have diabetes or sugar diabetes?” (of any form or multiple forms of diabetes). For this study, the participants who answered “borderline” or “yes” were considered diabetics. Those who answered “no” were considered non-diabetics.

### 2.5. Measures: Additional Covariates

Obesity data from the NHANES dataset were subdivided into four categories according to body mass index (BMI) derived from measured height and weight. The categories were as follows: participants with BMI < 25 were considered normal weight; participants with a BMI = 25–29 were overweight; participants with a BMI = 30–39.9 were considered obese; and participants with a BMI > 40 were considered severely obese. For the multivariate models, obesity was dichotomized and considered present for a BMI ≥ 30 and considered absent for the rest. The data included health-related behaviors, such as cigarette smoking (categorized as “Never” (<100 cigarettes in life), “Former” (>100 cigarettes but not currently smoking), and “Current” (smoking “some days” or “every day”)). Demographic characteristics were also assessed as covariates (e.g., age, gender, and ethnicity) Ethnicity was divided into “Non-Hispanic White”, “Non-Hispanic Black”, “Hispanic”, and “Other”. Finally, the education-level data were subdivided into trichotomous indicators as “Completing some high school” versus “High school graduate” versus “Some college or above”. Income level was determined using the family monthly poverty index, where a value of <2.00 indicated low income.

### 2.6. Statistical Analysis

We weighted the demographic variables to approximate distributions in the USA using the provided sample weights to account for the oversampling of older people, non-Hispanic Black individuals, and individuals of Mexican-American ethnic origin in the NHANES survey. We described continuous variables as the means ± standard deviations (SDs) and assessed the distributions of values using the Shapiro–Wilk test. Categorical variables were expressed as percentages and analyzed using chi-squared tests. Analysis was performed using Cox proportional hazards, and separate models for CRC versus all-cause mortality were run by SI after adjusting for sociodemographic and health factors. Statistical analyses were conducted using the SAS System for Windows (release 9.3; SAS Institute Inc., Cary, NC, USA). All analyses included sample weights that accounted for the unequal probabilities of selection and nonresponse. All variance calculations incorporated the sample weights and accounted for the complex sample design using Taylor series linearization. All significance tests were two-sided using a value of *p* < 0.05.

## 3. Results

After combining the data from the NHANES 2005–2018, we had an unweighted sample size of 34,276 individuals (weighted *n* = 143.3 million) aged 20 years and older with complete medical examination data. Table 1 displays the demographic characteristics of the sample. Approximately 54.9% were women, 67.9% were non-Hispanic White, and their mean age was 47.5 years. The average duration of follow-up for all study participants was 7.5 years. Data on the distribution of demographic characteristics of the study participants were stratified by CRC diagnosis using bivariate analysis. Individuals with CRC were statistically significantly more likely to be older, White, current or former smokers, diabetic, have reported suicidal ideation, have a lower level of education, and have a history of CVD, diabetes, or hypertension.

**Figure 1:**
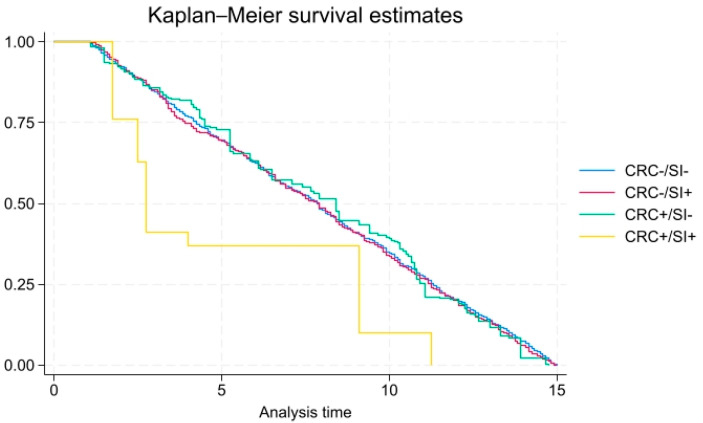
All-cause mortality among individuals with and without CRC/SI (x-axis: time in years; y-axis: overall survival probability).

For all-cause mortality, the unadjusted hazard ratio (HR) among those with CRC irrespective of SI status was 5.44 (95% confidence interval (CI), 4.32–6.84, *p* < 0.01, not shown in Table 2). When adjusted for health and demographic variables, the risk of mortality among those with CRC irrespective of SI was still significantly elevated (HR = 1.39, 95% CI = 1.15–1.70). The adjusted HR was statistically significantly elevated more than five times (5.40 (CI 1.53–19.02, *p* < 0.01)) among individuals who had a history of CRC and had SI. Among people with CRC only (HR = 1.37, 95% CI= 1.13–1.67, *p* < 0.05) or SI only (HR = 1.42 95% CI = 1.16–1.75), the adjusted risk of mortality was also significantly elevated, but lower than mortality risk among individuals with both CRC and SI. As shown in Figure 1, there was a higher proportion of all-cause mortality over time among individuals with both SI and CRC than each variable independently. For instance, at 3 years, the mortality rate was 60% for individuals that had both a CRC and SI, compared the rate for each variable individually (CRC: 80% and SI: 80% mortality). There was also interactive effect between poverty and CRC as it related to all-cause mortality.

## 4. Discussion

In this nationwide study, we found that CRC and SI are, on their own, associated with a higher risk of mortality. However, the combined effect of having both CRC and SI on mortality far exceeded the effect of having one of these. A study by Jonson et al. [24] found that people with passive SI, defined as wishing to die, had twice the likelihood of all-cause non-suicide mortality, which may explain the increase in all-cause mortality among those with CRC if they also have SI. Also, certain markers of inflammation (e.g., CRP, IL-6) are elevated in chronic diseases, such as cancer, and have a relationship with poor mental health [17,18,25]. Several other studies have found a high prevalence of depression and increased inflammatory markers among people with CRC. These are major risk factors for suicide and mortality from other causes [21,25,26]. Also, biological pathways (e.g., cytokine dysregulation) or psychological mediators (e.g., hopelessness) are common pathways that can link CRC and SI. Researchers also found that the incidence of suicide was nearly doubled in cancer patients in comparison to the general population, with variations depending on the anatomic site of cancer.

Another major finding from this large nationally representative study was that individuals with CRC (without SI) had higher mortality, which aligns with findings from other studies. Compared to those without CRC, individuals with CRC suffer a higher risk of mortality and morbidity [13,14,15,27]. Furthermore, our analysis is the first in the U.S. to report that Americans with both SI and CRC are likely to experience increased mortality compared to those with either condition alone, creating a synergistic effect on overall mortality. In a Swedish cohort, researchers found that the risk of suicide was double that in the control group after CRC surgery [28]. In a different study, suicide risks associated with age at diagnosis, cancer stage, and cancer site were time-dependent [29]. Specifically, higher suicide risks were associated with the first two years of cancer diagnosis and an advanced cancer stage at diagnosis. Even after controlling for psychiatric conditions, studies have found that after a digestive tract cancer diagnosis, individuals have a higher risk of suicide mortality, with the risk varying based on the anatomic site of the cancer [25,26,27,28,29,30,31,32].

Lastly, a major finding of our analysis is that there was a higher prevalence of SI among CRC survivors. Additionally, an incidental finding is that poverty interacted with CRC, causing higher levels of mortality than in people who only had CRC. In a large meta-analysis conducted in 2020, Amiri et al. [31] found that the suicide rate for CRC had an increased standardized mortality ratio (SMR = 1.6). Sun et al. [21], in a Taiwanese study, found that the risk of suicide attempts was double among CRC patients compared to the controls. They also found that all demographics had increased risk, but the findings were limited to a short follow-up time. Studies have also shown that nearly a quarter or more of digestive tract cancer patients experience severe psychological distress, but most do not receive quality or timely mental health services or referrals for behavioral healthcare [30,31,32,33,34].

### 4.1. Recommendations

Mental illness and psychological distress screening for CRC is recommended due to the effect of CRC diagnosis on quality of life and survival and to improve prognosis [27,28,29,30,31,32,33,34,35,36]. Screening for mental disorders, such as depression and anxiety, can help prevent acute outcomes, such as suicide or SI. Patient and caretaker education is of the essence to recognize warning signs and symptoms of mental illnesses and psychiatric emergencies among people with CRC or any other cancer [25,26,27,28,29,30]. Most importantly, there is also a need for the involvement of a patient-centered medical team that is collaborative and multidisciplinary [33,34,35,36]. Future studies should also have longer follow-up times for cancer studies and psychological distress to assess outcomes, such as suicide and mortality, from other causes. Further studies and interventions are needed to target and identify these high-risk groups vulnerable to suicide. Finally, there are unmeasured confounders (e.g., social support, treatment adherence) that could be assessed in future research [32,33,34,35,36].

### 4.2. Limitations

The results suggest that patients with CRC who experience SI have a higher probability of death than those individuals with CRC or suicidal ideation alone. However, given the cross-sectional nature of the NHANES, a cause-and-effect relationship between suicidal ideation and mortality among those with CRC cannot be established. Also, we were unable to ascertain if SI came before or after the diagnosis of CRC or vice versa because the time of diagnosis is not available in the NHANES data. An assumption can be made that a diagnosis of SI may have been present earlier due to the wording of the question asking about the past two weeks (i.e., preceding CRC diagnosis). Additionally, there is a potential for recall bias and social desirability bias. Another limitation of this study was that we only studied all-cause mortality, not CRC-related or SI-related mortality. In addition, SI was assessed via a single survey question, potentially oversimplifying its complexity. However, the SI question from the PHQ-9 has been used by previous researchers. Similarly, CRC diagnosis relies on self-reporting, introducing potential recall bias (e.g., underreporting in asymptomatic cases).

## 5. Conclusions

In this nationally representative, multiethnic study, we found that people with a history of CRC are at higher risk of mortality than the general population. More importantly, we determined in unadjusted and adjusted models that SI and CRC combined have worse mortality outcomes than CRC alone. We also found that poverty and CRC has an interactive effect on all-cause mortality, highlighting the importance of assessing social determinants of health. Depression and suicide screening should be implemented by oncologists and healthcare practitioners involved in the care of cancer patients. Further research is needed to determine the longitudinal relationship in the connection between mental health and physical health.

## Figures and Tables

**Table 1:** Characteristics of study participants aged 20 and over with CRC and suicidal ideation.

Characteristics	Total Population (*n* = 34,276)	CRC (+) (*n* = 208)	CRC (−) (*n* = 34,015)
Smoking Status *			
Never smoked	54.8 (53.8–55.9)	45.5 (27.7–63.3)	54.9 (53.9–55.9)
Formerly smoked	25.0 (24.3–25.7)	40.4 (24.9–56.0)	24.2 (24.2–25.6)
Currently smoke	20.2 (19.5–20.9)	14.1 (10.0–18.1)	20.2 (19.5–20.9)
Obesity (yes, 95%CI)			
Obese BMI ≥ 30	37.6 (37.0–38.3)	42.6 (31.5–53.7)	37.6 (36.9–38.3)
Hypertension (yes, 95% CI) **	32.2 (31.5–32.8)	67.9 (62.8–73.1)	32.0 (31.3–32.7)
Diabetes (yes, 95% CI) **	11.8 (11.3–12.3)	25.8 (19.7–32.0)	11.8 (11.3–12.2)
Cardiovascular Disease (yes, 95% CI) **	8.6 (8.3–9.0)	23.2 (17.7–28.6)	8.5 (8.2–8.9)
Suicidal Ideation **			
Not at all	96.7 (96.5–96.9)	93.4 (86.1–97.0)	96.7 (96.5–96.9)
Several days	2.3 (2.2–2.5)	2.0 (0.5–8.2)	2.3 (2.2–2.5)
More than half the days	0.5 (0.4–0.6)	2.0 (0.9–4.5)	0.5 (0.4–0.6)
Nearly every day	0.4 (0.3–0.5)	2.6 (0.6–10.8)	0.4 (0.3–0.5)
Age (years, SE) **	47.5 (0.19)	66.7 (1.64)	47.4 (0.19)
Gender (Male %, 95% CI)	48.7 (48.3–49.1)	46.1 (36.4–55.9)	48.7 (48.3–49.1)
Ethnicity **			
Non-Hispanic White	67.9 (65.9–69.8)	82.2 (77.4–87.0)	67.8 (6.3–67.7)
Non-Hispanic Black	11.0 (9.7–12.3)	10.1 (5.6–14.6)	11.0 (9.7–12.3)
Hispanic	13.8 (12.0–15.6)	5.1 (3.0–8.5)	13.9 (12.1–15.7)
Other	7.3 (6.2–8.6)	2.6 (1.2–5.5)	7.3 (6.2–8.6)
Education Level *			
Some high school	17.0 (15.8–18.3)	23.9 (18.3–29.5)	17.0 (17.9–20.0)
High school graduate	22.2 (21.4–23.1)	19.1 (12.9–25.3)	22.3 (21.4–23.1)
Some college and beyond	60.7 (59.7–62.5)	57.0 (49.4–64.7)	60.8 (59.0–62.5)
PIR < 2 (yes, 95% CI)	13.8 (13.1–14.4)	13.7 (10.5–16.9)	13.8 (13.1–14.4)
All deaths (N, %) **	3609 (7.7)	83 (34.6)	3526 (7.6)

Note. * *p* < 0.05 ** *p* < 0.01.

**Table 2:** Risk of All-Cause Mortality Based on SI or CRC Status Among Study Population.

	Total Population HR (95%CI) CRC vs. No-CRC	SI Without CRC HR (95%CI)	CRC Without SI HR (95%CI)	Both CRC and SI HR (95%CI)
CRC or SI	1.39 (1.15–1.70) *	1.42 (1.16–1.75) *	1.37 (1.13–1.67) *	5.40 (1.53–19.02) **
Smoking Status				
Never smoked	Ref.	Ref.	Ref.	Ref.
Formerly smoked	1.20 (1.07–1.34) *	1.20 (1.07–1.34) **	1.21 (1.09–1.34) **	0.83 (0.38–1.83)
Currently smoke	2.25 (2.03–2.49) **	2.25 (2.03–2.49) **	2.25 (2.04–2.49) **	1.65 (0.97–2.81)
Obesity (yes vs. no)	0.97 (0.89–1.06)	0.97 (0.89–1.06)	0.97 (0.89–1.06)	1.04 (0.72–1.50)
Hypertension (yes vs. no)	1.23 (1.12–1.34) **	1.23 (1.12–1.35) **	1.22 (1.11–1.33) **	1.62 (1.02–2.57) *
Diabetes (yes vs. no)	1.47 (1.37–1.58) **	1.48 (1.36–1.56) **	1.48 (1.38–1.59) **	1.15 (0.81–1.62)
CVD (yes vs. no)	1.71 (1.54–1.89) **	1.71 (1.55–1.87) **	1.74 (1.58–1.92) **	1.01 (0.71–1.44)
Age	1.09 (1.08–1.10) **	1.09 (1.08–1.10) **	1.09 (1.08–1.10) **	1.10 (1.07–1.13) **
Gender (ref. female)	1.31 (1.21–1.41) **	1.31 (1.21–1.41) **	1.32 (1.21–1.44) **	1.22 (0.79–1.90)
Ethnicity				
Non-Hispanic White	Ref.	Ref.	Ref.	Ref.
Non-Hispanic Black	1.00 (0.91–1.11) *	1.01 (0.91–1.12)	1.01 (0.92–1.12)	0.85 (0.51–1.41)
Hispanic	0.64 (0.55–0.75) **	0.64 (0.54–0.74) **	0.66 (0.57–0.78) **	0.36 (0.22–0.60) **
Other	0.68 (0.54–0.85) *	0.68 (0.55–0.86)	0.69 (0.55–0.87) *	0.37 (0.17–0.80) *
Education Level				
Some college or beyond	Ref.	Ref.	Ref.	Ref.
Some high school	1.30 (1.07–1.59) *	1.33 (1.10–1.60) *	1.31 (1.07–1.59) *	1.13 (0.81–1.58)
High school graduate	1.22 (1.10–1.36) **	1.23 (1.11–1.37) **	1.24 (1.10–1.39) **	0.93 (0.62–1.41)
PIR ≥ 2	1.72 (1.53–1.94) **	1.70 (1.51–1.90) **	1.69 (1.52–1.88) **	2.16 (1.54–3.02) **

Note. * *p* < 0.05 ** *p* < 0.01. HR (95%CI) indicates hazard ratios with 95% confidence intervals for the outcome (i.e., mortality). Ref. indicates the reference group among each variable for comparison with other groups. CRC = colorectal cancer, SI = suicidal ideation. CVD = cardiovascular diseases.

## Data Availability

The data are available from the CDC website by the National Center for Health Statistics.
